# Supplementary material for: Extracting lung function measurements to enhance phenotyping of chronic obstructive pulmonary disease (COPD) in an electronic health record using automated tools
Source: PLoS One. 2020 Jan 16;15(1):e0227730. doi: 10.1371/journal.pone.0227730 (PMC6964890; doi:10.1371/journal.pone.0227730)
Supplement: S1 Data code — (PDF) [file pone.0227730.s001.pdf]

S1 Data code. SQL script used for extracting FEV1 values from text notes in VA clinical notes.

```
/**script for extracting FEV1 values from report text**/

use VACS_TIU

declare @keyword varchar(30)='FEV'

if object_id('tempdb..#t1') is not null

        Drop table  #t1

/**identify documents that contain 'FEV' in the text**/

select TIUDocumentSID, reporttext

into #t1

from [VACS_TIU].[dbo].[PFT_TIU]

where contains(reporttext, @keyword)
```

```
/**extract snippets of 20 characters long starting at pos where "FEV" occurs -- there may be multiple occurrences**/

update #t1

set reporttext=Replace(replace(replace(cast(reporttext as varchar(max)) ,char(13),''), char(10),''),char(9),'')



if object_id('tempdb..#t2') is not null

	drop table #t2



;with T2 as (
   select 0 as row, tiudocumentsid,charindex(@keyword, reporttext, 0) pos, reporttext,substring(reporttext, charindex(@keyword, reporttext),20) snippet
	from #t1
   union all
   select pos + 1 as row,tiudocumentsid, charindex(@keyword, reporttext, pos + 1), reporttext,substring(reporttext, charindex(@keyword, reporttext,pos+1),20) snippet
   from T2
   where pos > 0
)

select * into #t2
from T2
order by tiudocumentsid




if object_id('tempdb..#t3') is not null
drop table #t3
```

```
/**process the snippets to find FEV patterns such as "FEVER", "FEV=", "FEV -1", "FEV- 1" and "FEV- 1." etc and extract numeric values right after such patterns**/


select #t2.*, fev1val=
	case upper(substring(snippet,1,6))
		when 'FEV- 1' then
			case  upper(substring(snippet,1,7))
			when 'FEV- 1.' then
				stuff(stuff(substring(snippet,6,4)+'x', patindex('%[0-9][^0-9.]%', substring(snippet,6,4)+'x') + 1, len(substring(snippet,6,4)), ''
			), 1, patindex('%[0-9]%', substring(snippet,6,4)) - 1, '')
			else
				stuff(stuff(substring(snippet,7,20)+'x', patindex('%[0-9][^0-9.]%', substring(snippet,7,20)+'x') + 1, len(substring(snippet,7,20)), ''
			), 1, patindex('%[0-9]%', substring(snippet,7,20)) - 1, '')
			end
		when 'FEV -1' then
			stuff(stuff(substring(snippet,7,20)+'x', patindex('%[0-9][^0-9.]%', substring(snippet,7,20)+'x') + 1, len(substring(snippet,7,20)), ''
			), 1, patindex('%[0-9]%', substring(snippet,7,20)) - 1, '')
		when 'FEV 1 ' then
			stuff(stuff(substring(snippet,7,20)+'x', patindex('%[0-9][^0-9.]%', substring(snippet,7,20)+'x') + 1, len(substring(snippet,7,20)), ''
			), 1, patindex('%[0-9]%', substring(snippet,7,20)) - 1, '')
		when 'FEV 1:' then
			stuff(stuff(substring(snippet,7,20)+'x', patindex('%[0-9][^0-9.]%', substring(snippet,7,20)+'x') + 1, len(substring(snippet,7,20)), ''
			), 1, patindex('%[0-9]%', substring(snippet,7,20)) - 1, '')
		when 'FEV 1,' then
			stuff(stuff(substring(snippet,7,20)+'x', patindex('%[0-9][^0-9.]%', substring(snippet,7,20)+'x') + 1, len(substring(snippet,7,20)), ''
			), 1, patindex('%[0-9]%', substring(snippet,7,20)) - 1, '')
		when 'FEV OF' then
```

```
                    stuff(stuff(substring(snippet,7,20)+'x', patindex('%[0-9][^0-9.]%', substring(snippet,7,20)+'x') + 1,
len(substring(snippet,7,20)), ''


                    ), 1, patindex('%[0-9]%', substring(snippet,7,20)) - 1, '')
          else
                    case upper(substring(snippet,1,5))
                    when 'FEVER' then NULL
                    when 'FEV 1' then
                              case upper(substring(snippet,1,8))
                              when 'FEV 1.0=' then


                                        stuff(stuff(substring(snippet,9,20)+'x', patindex('%[0-9][^0-9.]%',
substring(snippet,9,20)+'x') + 1, len(substring(snippet,9,20)), ''


            ), 1, patindex('%[0-9]%', substring(snippet,9,20)) - 1, '')
                              else
                                        case upper(substring(snippet,1,6))
                                        when 'FEV 1.' then
                                                  stuff(stuff(substring(snippet,5,20)+'x', patindex('%[0-9][^0-9.]%',
substring(snippet,5,20)+'x') + 1, len(substring(snippet,5,20)), ''
            ), 1, patindex('%[0-9]%', substring(snippet,5,20)) - 1, '')
                                        else
                                                  stuff(stuff(substring(snippet,6,20)+'x', patindex('%[0-9][^0-9.]%',
substring(snippet,6,20)+'x') + 1, len(substring(snippet,6,20)), ''


            ), 1, patindex('%[0-9]%', substring(snippet,6,20)) - 1, '')
                                        end
                              end
                    else
                              case upper(substring(snippet,1,4))
                              when 'FEV=' then
                                        stuff(stuff(substring(snippet,5,4)+'x', patindex('%[0-9][^0-9.]%',
substring(snippet,5,4)+'x') + 1, len(substring(snippet,5,4)), ''
```

```
), 1, patindex('%[0-9]%', substring(snippet,5,4)) - 1, '')
when 'FEV ' then
stuff(stuff(substring(snippet,5,4)+'x', patindex('%[0-9][^0-9.]%', substring(snippet,5,4)+'x') + 1, len(substring(snippet,5,4)), ''
), 1, patindex('%[0-9]%', substring(snippet,5,4)) - 1, '')
else
stuff(stuff(substring(snippet,6,20)+'x', patindex('%[0-9][^0-9.]%', substring(snippet,6,20)+'x') + 1, len(substring(snippet,6,20)), ''
), 1, patindex('%[0-9]%', substring(snippet,6,20)) - 1, '')
end
end
end

into #t3

from #t2

where pos>0

order by tiudocumentsid,pos

if object_id('tempdb..#t4') is not null

drop table #t4

select *, val=

case

when isnumeric(fev1val)>0 then fev1val

end
```

```
into #t4

from #t3

/**put the processed results with the extracted FEV values in a persistent table**/

if object_id('VACS_TIU.dbo.keipftfev') is not null
        drop table [VACS_TIU].[dbo].[keipftfev]

SELECT

    [tiudocumentsid]

    ,[pos]

    ,[reporttext]

    ,[snippet]

    ,[fev1val]

    ,[val]

          ,cast(val as float) as val2

          into [VACS_TIU].[dbo].[keipftfev]

from #t4
```

```
/**a query to retrieve a subset -- e.g., extracted FEV values between 0.5 and 5.5**/

if object_id('VACS_TIU.dbo.keipftfevsub') is not null
	drop table [VACS_TIU].[dbo].[keipftfevsub]

select t2.scrssn, t2.referencedate,t1.[tiudocumentsid],t1.[pos],t1.[reporttext],t1.[snippet],t1.[fev1val],t1.[val2]

into [VACS_TIU].[dbo].[keipftfevsub]

from [VACS_TIU].[dbo].[keipftfev] t1 inner join [VACS_TIU].[dbo].[pft_tiu] t2

on t1.tiudocumentsid=t2.tiudocumentsid

where t1.val2>=0.5 and t1.val2<=5.5 and t1.fev1val !='1'



if object_id('VACS_TIU.dbo.keipftfevsubct') is not null

	drop table [VACS_TIU].[dbo].[keipftfevsubct]

select scrssn,tiudocumentsid,count(*) as ct

into [VACS_TIU].[dbo].[keipftfevsubct]

from

[VACS_TIU].[dbo].[keipftfevsub]

group by scrssn,tiudocumentsid
```

```
select t1.scrssn,t1.tiudocumentsid,t1.referencedate,t1.pos,t1.reporttext,t1.snippet,t1.val2 from
[VACS_TIU].[dbo].[keipftfevsub] t1 inner join [VACS_TIU].[dbo].[keipftfevsubct] t2

on t1.scrssn=t2.scrssn and t1.tiudocumentsid=t2.tiudocumentsid

where t2.ct>1

order by scrssn,tiudocumentsid
```
